# Diagnosis challenges in CHARGE syndrome: A novel variant and clinical description

**DOI:** 10.1016/j.heliyon.2024.e28024

**Published:** 2024-03-15

**Authors:** Samantha Saenz Hinojosa, Carlos Reyes, Vanessa I. Romero

**Affiliations:** a School of Medicine, Universidad San Francisco de Quito, Quito, Ecuador; b Genetics Department, Hospital de Especialidades Eugenio Espejo, Quito, Ecuador

**Keywords:** CHARGE syndrome, Genetic testing, Misdiagnosis, CHD7, Noonan syndrome

## Abstract

**Introduction:**

In resource-limited settings, patients with uncommon phenotypes often face prolonged diagnostic journeys and potential misdiagnoses. Coloboma, heart defects, atresia choanae, restricted growth and development, genital and ear abnormalities syndrome (CHARGE) syndrome, a congenital condition affecting various body parts such as the heart, ears, eyes, and genitals, exemplifies this challenge.

**Case presentation:**

We present the case of a 21-year-old male patient from Ecuador who exhibited hypogonadism, facial deformities, and stunted growth. Due to the scarcity of genetic specialists and limited access to genetic testing in Ecuador, the patient received a misdiagnosis of Noonan syndrome. However, a correct diagnosis of CHARGE syndrome was ultimately reached after eight years, facilitated by genetic sequencing that identified a novel mutation in the Chromodomain helicase DNA binding protein 7 gene.

**Conclusion:**

This case highlights the critical role of meticulously assessing patients' symptoms and emphasizes the necessity for enhanced collaboration among physicians and researchers. Such efforts are pivotal in advancing healthcare access and equity for individuals in developing nations.

## 1 Introduction

CHARGE syndrome (Coloboma, heart defects, atresia choanae, restricted growth and development, genital and ear abnormalities syndrome) (OMIM 214800) encompasses a distinctive set of features including coloboma (80–90%), heart defects (75–85%), choanal atresia (50–60%), growth, and neurodevelopmental delay (70%), morphological central nervous system abnormality, external genital hypoplasia (70% in males and 30% in females), abnormalities of the outer ear, and hearing impairment (>90%) ([1]. Pagon et al. established major and minor criteria for CHARGE syndrome diagnosis in 1998, requiring the presence of four major and three minor criteria for a definitive diagnosis. Furthermore, a diagnosis may be considered when one or two major criteria and multiple minor criteria coexist.

CHARGE syndrome, an infrequent condition detected in neonates, presents a diagnostic challenge based only on clinical findings. Diagnostic protocols include cytogenetic testing for integrity in chromosomes 22, 14, and 9, in addition with genetic assays targeting stop or frameshift mutations within the chromodomain helicase DNA binding protein 7 (CHD7) gene resulting in a null protein [2]. Although there is currently no cure for CHARGE syndrome, individuals require intensive medical management and surgical interventions focusing on appearing symptoms to allow for an average life expectancy [2].

Our clinical description focuses on a 21-year-old male patient. Initially misdiagnosed with Noonan syndrome at the age of 13, he received an accurate diagnosis of CHARGE syndrome through genetic sequencing at 21. Despite evident symptoms such as hypogonadism, facial dysmorphia, and stunted growth, clinical genetic evaluation was deferred until adulthood. This case highlights the complex nature of diagnosing rare disorders, particularly when they exhibit uncommon phenotypes, potentially leading to long diagnostic journeys.

### 1.1 Clinical report

**Fig. 1:**
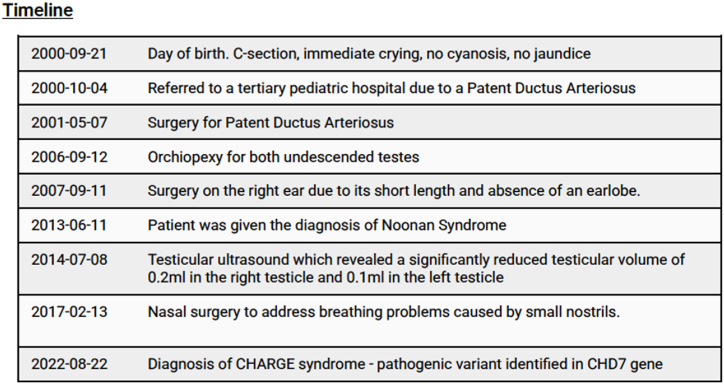
Historical and current information from this episode of care organized as a timeline.

We present a case involving an individual with CHARGE syndrome born as a full-term twin to a 35-year-old mother, with no documented complications during pregnancy. Notably, the individual's twin sibling exhibits good health and lacks any related phenotypic manifestations. Fig. 1 provides a chronological overview of the medical history.

At 15 days of age, the individual with CHARGE syndrome was referred to a tertiary pediatric hospital due to a Patent Ductus Arteriosus (HP:0011648), which was surgically corrected at eight months of age. At age 6, he underwent orchiopexy procedure for cryptorchidism (HP:0000028). At age 7, the individual with CHARGE syndrome underwent right ear surgery due to hypoplasia of the earlobe (HP:0009906). During early childhood (age not specified), the individual with CHARGE syndrome experienced hearing loss in the left ear, with a 25% hearing impairment (HP:0000365).

**Fig. 2:**
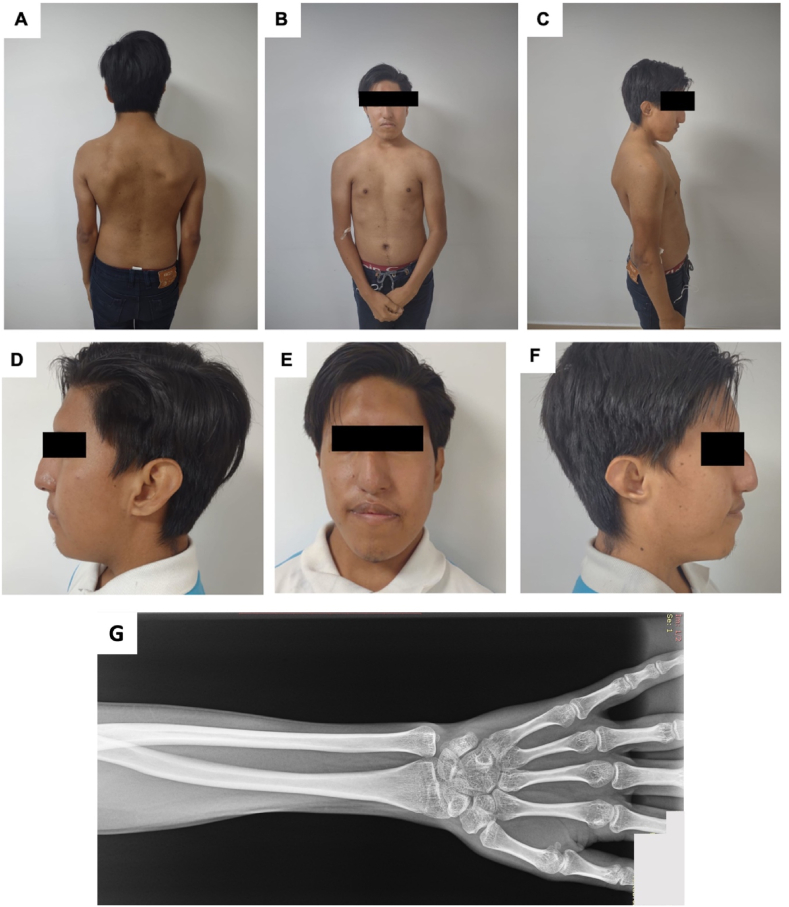
Patient at 21 years old. (A)(B)(C) Asymmetrical thorax with asymmetry of the shoulders, prominent scapula and scoliosis. (D)(E)(F) Facial characteristics: facial asymmetry, a square face with a prominent forehead, and a prominent nasal bridge. G)Wrist RX of patient at 21 years old. Delayed bone age and decreased bone mineral density for patient's age.

A testicular ultrasound at age 14 revealed significantly decreased testicular volume (HP:0008734) – 0.2ml in the right testicle and 0.1ml in the left testicle, substantially below the normal range of 4ml. The individual with CHARGE syndrome had decreased serum testosterone (HP:0040171), ranging from 107 ng/dl to 200 ng/dl (normal values 270–1070 ng/dL based on age), which improved to 430 ng/dl following oral testosterone therapy. Laboratory analyses reported low free T4 (Thyroxine) levels (0.87 ng/dL- normal values 0.9–2.3ng/dL) while maintaining a normal TSH (Thyroid stimulating hormone) −1.58 ng/dL. The individual with CHARGE syndrome echocardiogram yielded normal results. Despite lacking the patient-provided spine X-ray, his medical record described reduced interdiscal space between C6–C7 and mild cervical and dorsal scoliosis (Fig. 2A–F). At age 17, nasal surgery was conducted to address respiratory difficulties attributed to narrow naris (HP:0009933). Ophthalmological assessments disclosed an inferior chorioretinal coloboma (HP:0031613), and bilateral horizontal nystagmus (HP:0000666), with the left eye displaying more pronounced effects. The individual with CHARGE syndrome medical history indicates a dysharmonic delayed bone age (HP:0005832) and reduced bone mineral density (HP:0004349), as shown in Fig. 2.

The individual with CHARGE syndrome underwent initial evaluation at a hospital within the Public Health System of Ecuador. However, it was only at the age of 21 that he underwent assessment by a geneticist from the Human Genetics Department at a private university in Ecuador. At 21 years old, he presents facial deformities (Fig. 2A–F), including asymmetry (HP:0000324), a square-shaped face (HP:0000321) with a prominent forehead (HP:0011220), and a wide nasal bridge (HP:0000431) (Table 1). Asymmetry of the thorax (HP:0001555) is discernible, characterized by a sprengel anomaly (HP:0000912) and the left shoulder positioned higher than the right. No abnormalities are evident in his extremities. Moreover, the individual has stature short stature (HP:0004322), measuring 159 cm, in contrast to the 165 cm average height for Ecuadorian men, as detailed in the ENSANUT-ECU, a Health and Nutrition Survey conducted by the Ecuadorian Ministry of Health [3]. The individual with CHARGE syndrome and the family mention that no other family member presents with genetic disorders and no history of a related phenotype.

**Table 1:** Criteria for diagnosis of CHARGE syndrome that are present in our patient.

Major Criterion (the four Cs)	Our patient
Coloboma	Coloboma of the inferior optic nerve, an inferior retinochoroid coloboma
Cranial nerve abnormalities - Sensorineural hearing loss due to abnormalities in cranial nerve VIII - Swallowing problems (cranial nerves IX/X) - Asymmetric facial palsy (cranial nerve VII) - Absent or reduced sense of smell (cranial nerve I)	Our patient presents with hearing loss in the left ear
Choanal atresia	–
CHARGE Ear (the typical is short and wide with little or no earlobe)	Patient was born with a short ear with no earlobe, which was surgical corrected at age 7-years-old.
**Minor Criterion**	**Our Patient**
Heart defects	Born with Patent Ductus Arteriosus corrected surgically at 8-months
Genital abnormalities	Hypogonadism
Developmental delay	–
Cleft lip and/or cleft palate	–
Tracheoesophageal Fistula/Esophageal atresia	–
Poor growth	Our patients heigh is under the average for the men in the same country
CHARGE face - Square face - Prominent forehead - Arched eyebrows - Large eyes - Occasional droopy eyelids - Prominent nasal bridge - Small nostrils - Prominent nasal columella - Flat midface - Small mouth - Small chin Face is often asymmetric	Asymmetrical face, square face, prominent forehead, prominent nasal bridge, small nostrils (surgically corrected at 17-years-old)
**Occasional Findings**	**Our Patient**
Kidney abnormalities	–
CHARGE hand - Small thumb - Broad palm with hockey stick palmar crease Short fingers	–
Thymic/parathyroid hypoplasia	–
General appearance: - Webbed neck - Sloping shoulders - Nipple anomalies	–
Abdominal defects: - Omphalocele - Umbilical hernia	–
Spine anomalies: scoliosis	Our patient presents mild cervical and dorsal scoliosis

The geneticist ordered the Congenital Heart Disease and CHARGE Panel, which analyzes 56 genes. The panel was conducted through a private laboratory located in the United States of America using Illumina technology. All targeted regions were sequenced with≥50x depth. The output reads are aligned to a reference sequence genome build GRCh37 (Genome Reference Consortium Human Build 37). The panel identified a heterozygous nonsense pathogenic variant in exon 30 of CHD7 gene (NM_017780.3 c.5897G > A (p.Trp1966*), pathogenic, CHARGE syndrome, autosomal dominant), which creates a premature translational stop signal (p.Trp1966*) in the CHD7 gene, which is expected to result in an absent or disrupted protein product. Loss of function variants in *CHD7* are known to be pathogenic [4,5]. Algorithms developed to predict the effect of sequence changes on RNA (Ribonucleic acid) splicing suggest that this variant may create or strengthen a splice site; hence the private laboratory report classified this variant as Pathogenic.

This variant is not present in the population database Genome Aggregation Database (gnomAD v2.1.1 no frequency) and is associated with autosomal dominant CHARGE syndrome. This variant was also not found in the NCBI RefSeq or Locus Reference Genomic (LRG) database. This premature translational stop signal has been observed in individuals with congenital heart disease [6]. Based on the Criteria for Classifying Pathogenic Variants from the joint consensus of the ACMG (American College of Medical Genetics) and AMP (American Association of Molecular Pathology), this variant is classified as a Pathogenic Variant complying with the characteristics for very strong evidence of pathogenicity PVS1 as it is a loss of function mutation and strong evidence of pathogenicity PS2 with confirmed maternity and paternity and both parents do not have any phenotype [7]. No other candidate variants were found.

The geneticist evaluated the individual with CHARGE syndrome after giving the correct diagnosis at 22 years old. The prognosis is favorable, as individuals are more vulnerable and at higher risk during the early years of life [8]. This case underscores the impact of a lack of clinical assessment in the younger years, which has contributed to the delayed the correct diagnosis. The follow-up plan involves an interdisciplinary team to offer a comprehensive and unified approach to address his needs: an otolaryngologist to focus on his hearing impairment, an ophthalmologist to monitor his coloboma, an endocrinologist to replace his testosterone well and monitor his thyroid function, bone density and overall bone health. Additionally, genetic counseling is incorporated into the holistic care plan. It is crucial to implement a cohesive approach to managing his symptoms, ensuring early identification of possible complications, and ultimately favoring his prognosis.

## 2 Discussion

This clinical description focuses on a 21-year-old Ecuadorian male who underwent evaluation at a genetic clinic after an eight-year interval following his initial misdiagnosis of Noonan syndrome at age 13. Physical examination using Blake criteria fits the diagnosis of CHARGE as depicted in Fig. 2 with criterion including coloboma, ear abnormalities, cranial nerve dysfunction (hypoplasia of the auditory nerve), cardiovascular malformations, hypogonadotropic hypogonadism, growth deficiency and distinctive face. A sequencing panel reported a novel pathogenic variant in the CHD7 gene.

The CHD7 gene, localized on chromosome 8q12.2, encodes the chromodomain helicase DNA binding protein 7 [9]. This multifunctional protein comprises various helicase family domains and is expressed across various embryonic regions, including the eye, inner ear, and brain [10]. It plays a pivotal role in chromatin organization and the modulation of gene expression through chromatin remodeling processes [10]. Deficiency in this protein leads to disruptions in chromatin remodeling and gene expression regulation, giving rise to the variable expressivity [11]. Various studies published CHD7's role in facilitating chromatin accessibility, histone modifications linked to active gene expression, and recruitment of RNA polymerase at enhancer regions implicated in brain tissue morphogenesis [12]. CHD7 also interacts with super-enhancer elements, functioning as a regulatory hub orchestrating the spatial and temporal dynamics of transcription factors in shaping neuroepithelial and central nervous system lineage identities [13]. Animal studies, particularly zebrafish and Xenopus models, underscore CHD7's significance in neural crest cell derived structures, reinforcing its relevance in CHARGE syndrome [14]. CHD7-null embryos exhibit conotruncal defects analogous to those evoked by neural crest cell dysfunction [15]. The mouse model mutation aligned with the human S834F mutation, unveiled that CHD7S824F/S824F embryos exhibited milder phenotypic manifestations compared to the CHD7-null embryos. This finding concurs with the human context, where the S834F heterozygous mutation corresponds to a less severe CHARGE phenotype [15]. Yan et al. suggested that nonsense and missense CHD7 mutations explains the phenotypic variances among individuals requiring personalized therapeutic interventions as a function of their mutation [15].

Given its multisystemic effects, diagnosing CHARGE syndrome poses challenges, leading to cases of underdiagnosis or misdiagnosis [16,17]. Within the CHARGE syndrome differential diagnosis, conditions like Abruzzo-Erickson syndrome, Kallmann syndrome, VACTERL (Vertebral defects, anal atresia, cardiac defects, tracheo-esophageal fistula, renal anomalies, and limb abnormalities) association, Kabuki syndrome, renal coloboma syndrome, Cat-eye syndrome, Joubert syndrome, and BOR syndrome feature (Table 2) [2]. Typically, Noonan syndrome is not included in the list of differential diagnoses and our patient did not meet the clinical criteria (Supplementary table 1). Noonan syndrome, an autosomal dominant genetic disorder, spans a spectrum of symptoms and physical attributes of varyin g severity, encompassing facial anomalies, neck abnormalities, short stature, skeletal malformations, congenital heart issues, vascular malformations, coagulation deficiencies, attention concerns, and mild intellectual impairment contingent on the affected gene [18].

**Table 2:** Differential diagnosis of CHARGE syndrome.

	Abbruzzo - Erickson Sydnrome	Kallmann Syndrome	VACTERL Association	Kabuki syndrome	Cat-eye syndrome	Joubert syndrome	BOR Syndrome
Cause	Mutation in the TBX22 gene	Mutations in more than 20 genes (ANOS1, CHD7, FGF8, FGFR1, PROK2, PROKR2)	Exact cause is unknown	Mutation in the KMT2D gene	Partial tetrasomy Chromosome 22 Partial trisomy Chromosome 22	Mutations in more than thirty genes related to structures of primary cilia	Mutation in the EYA1 (BOR1, BOS2), SIX5 (BOR2) and SIX1(BOR2, BOS3) genes
Symptoms							
Facial	Cleft palate Flat Face Macrotia Malar Flattening Abnormal palate morphology Chorioretinal coloboma Coloboma Iris Coloboma Dimple chin Epicanthus Microcornea	Cleft lip and palate Eye movement abnormalities Dental abnormalities	Facial asymmetry (hemifacial microsomia) Abnormal shape and size of ears	Palpebral fissures Everted lower eyelids Promient eyelashes broad nose with a flattened tip Misshaped ears Blue sclerae Ptosis Strabismus Cleft Palate Micrognathia	Ocular coloboma Minor ear defect preauricular skin tags or pits Microtia Strabismus Unilateral microphthalmia Aniridia Cleft Palate Ocular hypertelorism Micrognathia	Abnormal eye and tongue movements Abnormal development of the retina Coloboma Nystagmus Strabismus Ptosis	Pits or outgrows of cartilage in the front of the outer ear Cupped or small outer ear Narrow slanted outer ear canal Long narrow face Cleft palate
Upper Body	Radioulnar synostosis Ulnar deviation of finger Brachydactyly	Lack of breast development Scoliosis	Costovertebral abnormalities Radial aplasia, radial hypoplasia, triphalangial thumb	Small and/or thin fingernails Fetal finger pads Brachydactyly Clinodactyly Scoliosis	Scoliosis Vertebral fusions Radial aplasia	Polydactyly	
Genitals	Coronal hypospadias Hypospadias Cryptorchidism	Lack of menstrual periods No development of sex characteristics in males at puberty	Hypospadias				
Internal Organs	Abnormal localization of kidney Atrial septal defect	Renal agenesis	Cardiac defects (ventricular septal defects) Tracheal-esophageal abnormalities (atresia, stenosis, fistula) Renal abnormalities (renal aplasia, renal dysplasia, renal ectopia, hydronephrosis)	Congenital Heart defects Renal dysplasia or hypopolasia Hydronephrosis Horseshoe kidneys	Congenital Heart defects (Total anomalous pulmonary venous return) Unilateral or bilateral renal hypoplasia Unilateral agenesis Supernumerary kidney	Kidney and/or liver abnormalities	Branchial fistula Kidney abnormalities (Unusually shaped to duplication, or absence)
Lower Extremities	Short toe Toe syndactyly		Lower limb malformations	Flat feet			
Senses	Sensorinerual hearing imparirment Conductive hearing impairment	Anosmia			Conductive Hearing loss		
Other	Short Stature	Short stature Intertility	Anal atresia	Short Stature Mild to moderate intellectual disability Seizures Anal atresia	Anal Atresia Short stature	Ataxia Hyperpnea Sleep apnea Encephalocele	Hearing Loss

The individual with CHARGE underwent misdiagnosis for more than eight years, a consequence of resource limitations in Ecuador and restricted genetic testing capabilities. Initially assessed by hospitals within the Public Health System of Ecuador, it is essential to note that the country's health system is divided into two sectors: public and private. Despite the legal mandate for universal health coverage, the reality reveals a fragmented health system marked by limited public resources and constrained medical care outside major urban centers [19,20]. Approximately 51% of the population in Ecuador is covered by the Public Health System, yet accessibility remains a challenge, particularly in rural areas [21]. This case report focuses on a individual with CHARGEhailing from a rural area in the coastal region of Ecuador, acknowledged as one of the nation's most impoverished areas. Consequently, a significant disparity exists in clinical assessment and evaluation during early developmental stages, with restricted testing and a dearth of specialists. The patient ultimately received a conclusive diagnosis upon evaluation by the Human Genetics Department at a private university in Ecuador.

Despite reaching adulthood, the individual with CHARGE syndrome requires regular appointments with his primary care doctor and specialists to ensure ongoing monitoring, symptom management, and the prevention of potential complications. Adults with CHARGE syndrome face an elevated risk of mortality due to factors such as infection, aspiration, and obstructive sleep apnea [8]. Furthermore, hypogonadotropic hypogonadism resulting from GnRH deficiency is a prevalent endocrine phenotype observed in 60–80% of individuals with CHARGE syndrome [22]; therefore, it is imperative for patients to consult with a specialist reproductive endocrinologist for fertility assessment and treatment. Additionally, individuals with CHARGE syndrome should be directed to genetic counseling to obtain information into the risk of their offspring inheriting this condition. This case underscores the crucial role of clinicians in meticulously evaluating all clinical symptoms exhibited by the patient and exploring a range of diagnostic options, including those related to rare genetic disorders [23].

Thoroughly conducted differential diagnosis analysis, complemented by interdisciplinary teamwork, plays a pivotal role in achieving precise diagnoses, especially in cases of complex and interrelated symptomatology. Additionally, there is a social and economic burden related to misdiagnosis and late diagnosis of rare diseases. An economic evaluation report mentions that the burden of rare diseases is approximately ten times higher than other diseases, and the lack of treatment is associated with a 21.2% increase in total costs per patient per year [23]. As happened with our patient, because of lack of economic resources the genetic exams were not performed early; therefore, the patient was misdiagnosed for over 8 years. In a different study, it is mentioned that many countries, including countries in Latin America, lack or have limited evidence of the economic impact of rare diseases [24]. Moreover, a misdiagnosis is associated with mental health problems, caregiving burdens, deteriorated health and quality of life [24], and the process of seeking a diagnosis has been reported as mentally and emotionally draining for patients and their families [[25], [26]].

The experiences detailed in this report offer a valuable lesson for physicians, emphasizing the importance of avoiding misdiagnoses to enhance patient outcomes. There is a pressing need for policy interventions to address the challenges faced by physicians in Latin American countries, particularly by promoting diagnostic networks and fostering collaboration. Furthermore, it is crucial to enhace the accessibility of genetic specialists and advanced testing, especially for individuals with rare genetic conditions. Addressing these challenges holds the potential to enhance diagnostic accuracy and elevate the overall standard of healthcare for patients. Healthcare initiatives should transcend geographic and socioeconomic barriers, ensuring equitable care for all individuals. A limitation identified in this case report is the absence of clinical assessment and information regarding the individual with CHARGE syndrome younger years.

In summary, we acknowledge the inherent challenges associated with diagnosing rare disorders, given the potential overlap of symptoms with other conditions. This complexity can result in misdiagnoses and, consequently, inappropriate treatments, as evidenced by the patient in this study. Furthermore, we emphasize the critical role of collaboration between medical practitioners and researchers in advancing healthcare access and ensuring equity for patients in developing nations.

## Funding

No funding was provided for this research.

## Availability of data and materials

All data generated or analyzed during this study are available from the corresponding author on reasonable request.

## Consent for publication and photography

Informed consent and consented to the publishing of all images, clinical data, and other data included in the manuscript was obtained from the participant.

## CRediT authorship contribution statement

**Samantha Saenz Hinojosa:** Writing – review & editing, Writing – original draft, Methodology, Investigation, Formal analysis, Data curation, Conceptualization. **Carlos Reyes:** Visualization, Conceptualization. **Vanessa I. Romero:** Writing – review & editing, Writing – original draft, Visualization, Validation, Supervision, Resources, Project administration, Methodology, Investigation, Funding acquisition, Formal analysis, Data curation, Conceptualization.

## Declaration of competing interest

The authors declare that they have no known competing financial interests or personal relationships that could have appeared to influence the work reported in this paper.
