# Supplementary material for: Diagnosis challenges in CHARGE syndrome: A novel variant and clinical description
Source: Heliyon. 2024 Mar 15;10(6):e28024. doi: 10.1016/j.heliyon.2024.e28024 (PMC10965510; doi:10.1016/j.heliyon.2024.e28024)
Supplement: Multimedia component 1 [file mmc1.docx]

| **Characteristic** | **Our Patient** |
| --- | --- |
| **Facial Features** - Eyes: wide set and down slanting with droopy lids - Ears: low-set, rotated backward - Nose: depressed at the top with a wide base and bulbous tip - Facial features: may appear coarse - Head: large with a prominent forehead and low hairline | -  -  -  -  + |
| **Heart disease** - Valve disorders (Pulmonary valve stenosis) - Hypertrophic Cardiomyopathy - Ventricular Septal defect - Pulmonary artery stenosis  - Aorta coarctation - Irregular Heart Rhythm | -  -  -  -  -  - |
| Growth Issues | + |
| **Musculoskeletal Issues** - Pectus excavatum - Pectus carinatum - Wide-set nipples - Short webbed neck - Deformities of the spine | -  -  -  -  + |
| Learning disabilities | - |
| **Eye conditions** - Strabismus - Myopia - Hyperopia - Nystagmus - Cataracts | -  -  -  +  - |
| Hearing Problems | + |
| Bleeding | - |
| Lymphatic Conditions | - |
| **Genital conditions**  - Testicles: undescended - Puberty delayed  - Fertility: not develop normally in male patients | +  -  Unknown |

**Supplementary Table 1.** Noonan syndrome characteristics present in our patient.

Noonan syndrome - Symptoms and causes - Mayo Clinic [Internet]. [cited 2023 Mar 13]. Available from: https://www.mayoclinic.org/diseases-conditions/noonan-syndrome/symptoms-causes/syc-20354422
